# Exploring the potentials of *Sesuvium portulacastrum* L. for edibility and bioremediation of saline soils

**DOI:** 10.3389/fpls.2024.1387102

**Published:** 2024-06-10

**Authors:** Wenbin Zhang, Dan Wang, Dingding Cao, Jianjun Chen, Xiangying Wei

**Affiliations:** ^1^Institute of Oceanography, College of Geography and Oceanography, Minjiang University, Fuzhou, China; ^2^Fuzhou Institute of Oceanography, Fuzhou, China; ^3^Mid-Florida Research and Education Center, Department of Environmental Horticulture, Institute of Food and Agricultural Sciences, University of Florida, Apopka, FL, United States

**Keywords:** ecdysteroids, salt tolerance, sea purslanes, sea vegetables, *Sesuvium portulacastrum*, sustainable agriculture

## Abstract

*Sesuvium portulacastrum* L. is a flowering succulent halophyte in the ice plant family Aizoaceae. There are various ecotypes distributed in sandy coastlines and salty marshlands in tropical and subtropical regions with the common name of sea purslane. These plants are tolerant to salt, drought, and flooding stresses and have been used for the stabilization of sand dunes and the restoration of coastal areas. With the increased salinization of agricultural soils and the widespread pollution of toxic metals in the environment, as well as excessive nutrients in waterbodies, *S. portulacastrum* has been explored for the desalination of saline soils and the phytoremediation of metals from contaminated soils and nitrogen and phosphorus from eutrophic water. In addition, sea purslane has nutraceutical and pharmaceutical value. Tissue analysis indicates that many ecotypes are rich in carbohydrates, proteins, vitamins, and mineral nutrients. Native Americans in Florida eat it raw, pickled, or cooked. In the Philippines, it is known as atchara after being pickled. *S. portulacastrum* contains high levels of ecdysteroids, which possess antidiabetic, anticancer, and anti-inflammatory activities in mammals. In this review article, we present the botanical information, the physiological and molecular mechanisms underlying the tolerance of sea purslane to different stresses, its nutritional and pharmaceutical value, and the methods for its propagation and production in saline soils and waterbodies. Its adaptability to a wide range of stressful environments and its role in the production of valuable bioactive compounds suggest that *S. portulacastrum* can be produced in saline soils as a leafy vegetable and is a valuable genetic resource that can be used for the bioremediation of soil salinity and eutrophic water.

## Introduction

**Figure 1:**
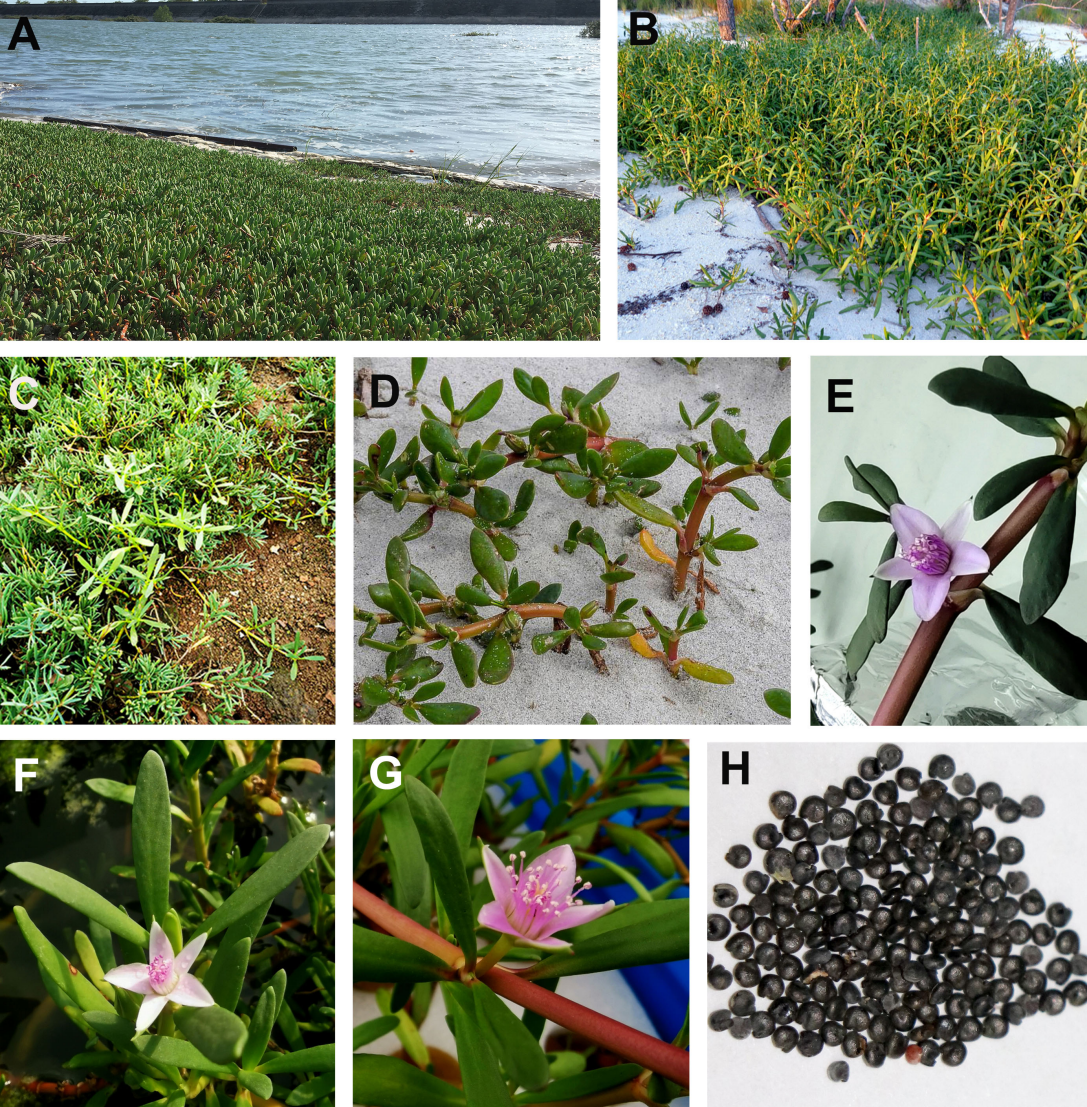
Growth habitats of *Sesuvium portulacastrum* ecotypes. **(A)** An ecotype with more succulent leaves grown by the beach. **(B)** A narrow-leaved ecotype with a light reddish stem grown in sandy soil. **(C)** An ecotype with light green, narrow leaves grown in rocky soil. **(D)** Plants are partially buried by sand. **(E–G)** Plants with purplish, whitish, and pinkish flowers. **(H)** Sea purslane seeds.

*Sesuvium* L. is a member of the family Aizoaceae that comprises around 18 species distributed in subtropical and tropical regions (Bohley et al., 2017; Sukhorukov et al., 2021). Phylogenetic analysis classifies them into African and American lineages. The African species include *Sesuvium congense*, *Sesuvium crithmoides*, *Sesuvium mesembryan-themoides*, *Sesuvium portulacastrum*, and *Sesuvium sesuvioides*, while representative American species include *Sesuvium curassavicum*, *Sesuvium humifusum*, *Sesuvium maritimum*, *Sesuvium mezianum*, *Sesuvium rubriflorum*, and *S. portulacastrum* (Sukhorukov et al., 2021). They are succulent, annual, or perennial herbaceous plants with papillate or prostrate glabrous stems, usually rooting at the nodes (**Figure 1**). Leaves occur opposite and are cylindrical, flattened with short petioles. Flowers are star-shaped and pink or purple, borne within the leaf axils. The fruit is a round capsule with 5–50 black seeds (Klak et al., 2003; Bohley et al., 2017; Klak et al., 2017a, Klak et al., 2017b; Sukhorukov et al., 2018). Almost all species in the African lineage are C_4_ plants, but some of the American species are derived from C_3_ plants, including *S. portulacastrum* L (Bohley et al., 2017; Winter et al., 2019). *S. portulacastrum* is a cosmopolitan species. The somatic chromosome number of *S. portulacastrum* varies depending on the ecotype. An early report indicated that the chromosome number of *S. portulacastrum* was 2*n* = 36 (Sharma and Bhattacharrya, 1956), but an ecotype in the Southeastern USA was found to be 2*n* = 16 (Di Fulvio, 1967; Bogle, 1970). An ecotype with oblong-oblanceolate leaves had 2*n* = 40 (Subramanian, 1988). However, the latest report suggested that the chromosome number of *S. portulacastrum* was 2*n* = 48 (Jena et al., 2003).

*S. portulacastrum* is distributed throughout coastal areas of the world, primarily on the continents of Africa, America, Asia, and Australia (Main and Waldrop, 2020). Due to its widespread distribution, various synonyms and common names have been given, including sea purslane and shoreline purslane. *S. portulacastrum* is a facultative halophyte and mainly inhabits sandy soils, coastal limestones, salt flats, and marshes. It is an important species that can colonize sandy beaches due to its stoloniferous, mat-forming growth habit, which acts to stabilize sand or protect sand from erosion. Thus, *S. portulacastrum* tolerates salt spray, sand scouring and burial, low soil nutrients, and high temperatures. It can accumulate a large amount of sodium (Na). In fact, Na at appropriate levels can increase the net photosynthetic rate of *S. portulacastrum* and enhance its growth (Peng et al., 2022).

Many ecotypes of *S. portulacastrum* or sea purslane plants are edible and are known as sea vegetables in the Caribbean, China, Europe, and India (Lokhande et al., 2009). They are rich in minerals and vitamins (Zeng et al., 2017; Main and Waldrop, 2020), and young shoots are collected and cooked or processed for consumption (Mathieu and Meissa, 2007; Gathii, 2013). The leaves have a high phenolic content and various compounds that possess pharmaceutical value (Chandrasekaran et al., 2011), and they have been used as traditional medicine to treat a number of human diseases, as well as plant pathogens and insect pests (Magwa et al., 2006; Youssif et al., 2019). In addition, sea purslane roots are often associated with several beneficial microbes that can promote plant growth (Yang et al., 2023). This important species, however, has been largely neglected or underutilized.

This review intended to summarize the recent progress in this unique species, including the physiological and molecular mechanisms underlying its tolerance to different stresses, its nutritional and pharmaceutical value, and the methods for its propagation and production. We believe that sea purslane is valuable and is an underexplored halophyte plant. Moreover, it can be easily grown in saline soils as a leafy vegetable and/or can be used for the bioremediation of the salinity of soils and waterbodies.

## Tolerance to abiotic and biotic stresses

Sea purslane plants show a wide range of adaptation to abiotic and biotic stresses. They can survive and thrive in soil irrespective of sea water spraying or flooding, thus are highly tolerant to salt and flooding stresses. Plants can also grow in dry sandy soils with low concentrations of mineral nutrients: they are tolerant to drought stress and nutrient deficiency. Thus far, a few reports have documented disease and pest problems in sea purslane plants, which are likely due to their resistance to several pathogens and insect pests. They can grow well not only in saline soils but also in soils without high levels of Na; therefore, they are known as facultative halophytes. Together, these characteristics enable *S. portulacastrum* to grow in different soils regardless of soil fertility and in different waterbodies as an aquatic plant.

### Salt tolerance

*S. portulacastrum* is remarkably tolerant to salinity. A study conducted in India showed that plant growth steadily increased when irrigated with nutrient solutions containing NaCl in the range from 100 to 500 mM, and the leaves with the highest total water content were those grown in 300 mM NaCl (Kannan et al., 2013). Similarly, the fresh weights of *S. portulacastrum* grown in a hydroponic system containing 100–500 mM Na were either equal to or were significantly higher than those grown in the same system without Na, and the root numbers of plants grown with 100 mM Na were similar to those produced without Na (Wang et al., 2022). Furthermore, Na has been found to be more effective than K in terms of cell expansion, leaf succulence, and shoot development (Wang et al., 2012). Thus, *S. portulacastrum* is considered a true salt-loving species, which possibly explains its ability to grow in saline seashores and wet sand accompanied by mangroves.

**Figure 2:**
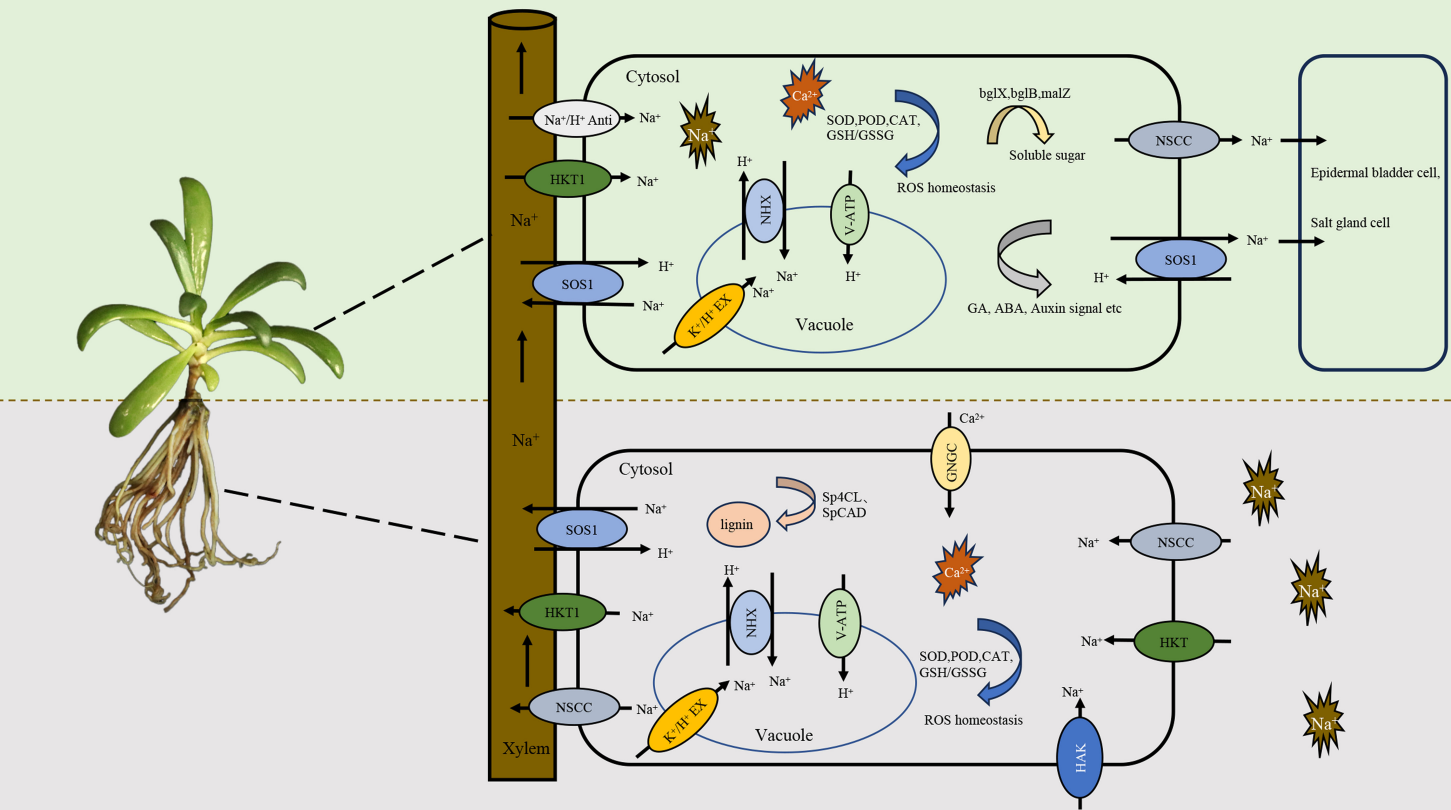
Proposed schematic model illustrating the absorption, transport, and compartmentation of Na in *Sesuvium portulacastrum* and plant overall tolerance to salt stress.

Recently, *S. portulacastrum* has been increasingly studied by physiological (Slama et al., 2006; Peng et al., 2019; Wang et al., 2023), proteomic (Cao et al., 2023), metabolomic (Kulkarni et al., 2022), and molecular (Chen Y. et al., 2022; Wang et al., 2022; Kulkarni et al., 2023) analyses of its tolerance to salt stress. This tolerance is not due to the avoidance of roots to Na or the restriction of Na in the roots; rather, it is attributed to the root absorption of Na and its transport from the roots to the shoots (Wang et al., 2012). In *S. portulacastrum*, the Na content increases with the increase of salinity (Yi et al., 2014; Muchate et al., 2016), and the Na concentration in the shoots is generally higher than that of K (Wang et al., 2022). These findings indicate that this species may have developed an integrative mechanism for salt tolerance during the course of plant adaptation to indigenous growth conditions. The exact mechanism, however, remains not fully understood. Based on the available information from recent studies of sea purslane and other plants, a schematic model for the absorption and transport of Na in *S. portulacastrum* is outlined in **Figure 2**. Na is absorbed by the roots through channels such as non-selective cation channels (NSCCs) and transporters including high-affinity potassium transporters (HKTs and HAKs) (Keisham et al., 2018; Wang et al., 2022). Na absorption triggers the activation of antioxidant enzymes, including catalase, peroxidase, and superoxide dismutase, to scavenge potential damage of reactive oxygen species (ROS) (Chen Y. et al., 2022) and the production of osmoprotectants, such as proline, flavonoids, and trehalose, to reduce the effects of Na (Wang et al., 2022; Cao et al., 2023). At the same time, genes related to the Na^+^/H^+^ antiporter (*NHX3*), sodium transporters, and vacuolar ATPase (V-ATPase) and salt overly sensitive 1 (*SOS1*) are induced (Nikalje et al., 2018; Wang et al., 2022). The absorbed Na is partially compartmentalized in vacuoles through NHX and K^+^/H^+^ exchange (Fan et al., 2017), which is mainly achieved by enhancing the V-ATPase activity rather than P-ATPase and H^+^-pyrophosphatase (Yi et al., 2014; Nikalje et al., 2018). The remaining cytosol Na is largely loaded into the xylem by NSCCs, *SOS1*, and HKTs, and then transported to the shoots through the transpiration stream (Wu, 2018; van Zelm et al., 2020). The Na in the xylem is loaded into the cells of the leaves through the activities of *SOS1*, the Na^+^/H^+^ antiporter, and the CCC co-transporter (Wu, 2018). Higher levels of Na in leaf cells quickly cause osmotic stress, depletion of K^+^, and the release of ROS. Leaves are more sensitive to Na than the roots as they can injure the photosynthetic apparatus, and the proteins in leaves have been found to be more sensitive to Na (Ding et al., 2022). In addition to the compartmentation of Na in the vacuole, it may be secreted through specialized salt glands. Although no reports are available on the secretion of Na in *S. portulacastrum*, this phenomenon has been documented in other halophytic plants (Wu, 2018; Spiekerman and Devos, 2020). At the same time, enzymatic and non-enzymatic antioxidants and osmoprotectants are produced to reduce the effects of Na (Zhou et al., 2023). It has been reported that the Na^+^/H^+^ antiporter gene *SpNHX1* from *S. portulacastrum* can enhance the salt tolerance of transgenic yeast (Zhou et al., 2018). Fan et al. (2018) reported that the overexpression of the plasma membrane H^+^-ATPase *SpAHA1* increased the salt tolerance of transgenic *Arabidopsis*. Gene ontology analysis showed that the proteins involved in ion binding, photosynthesis, proton transport, and ATP synthesis were overexpressed in salinity stress and that Na^+^/H^+^ reverse transporters and several ATP synthase subunits were activated under high salinity (Yi et al., 2014; Wang et al., 2022). Proteomic analysis showed 47 and 248 differentially expressed proteins (DEPs) in the shoots and roots, respectively, and a series of metabolites were differentially expressed in salt-stressed plants, with over 20% of these related to phenolic acid metabolism (Cao et al., 2023). Nevertheless, with continuous research on *S. portulacastrum*, our understanding of its tolerance to salt stress will be improved, and this schematic model will then be revised.

### Drought and flooding tolerance

Drought and flooding are two opposite stresses related to plant responses to the availability of water. As a halophyte, *S. portulacastrum* is highly tolerant to drought since it can grow on rocky shores, shell beaches, and coral cays or sandy beaches with a limited water supply (Lonard and Judd, 1997; Lokhande et al., 2009, Lokhande et al., 2011). On the other hand, this species can grow in seashore and wetland habitats, such as salt marshes and coastal zones. Thus, *S. portulacastrum* tolerates both drought and flooding.

Drought stress generally stimulates plants to produce signaling molecules, including abscisic acid (ABA), inositol-1,4,5-triphosphate (IP3), Ca^2+^, and cyclic adenosine 5′-diphosphate ribose (cADPR). The signals trigger the expression of a series of genes to produce stress-protectant metabolites, such as proline, glycine betaine, trehalose, and soluble sugars, and activate the plant antioxidant systems to sustain redox homeostasis (Ramani et al., 2006; Slama et al., 2006; Lokhande et al., 2011; Gupta et al., 2020; Yang X. et al., 2021). Drought stress has been shown to reduce the growth of *S. portulacastrum* by decreasing the leaf relative water content and increasing the proline, K, and Na contents, but rewatering stressed plants restored the plant growth, suggesting that *S. portulacastrum* retains its growth potential and nutrient absorption during drought stress, enabling it to quickly reestablish growth activity once water becomes available (Slama et al., 2006). After 5 days of drought stress, *S. portulacastrum* was able to allow more than 80% of stomatal opening (Meetam et al., 2020). Glycine betaine (GB), a quaternary amine with a zwitterionic nature, functions as an osmotic regulator under environmental stresses, including drought stress (Chen and Murata, 2008). The overexpression of *SpBADH*, a key gene in GB biosynthesis in *S. portulacastrum*, increased the tolerance of *Arabidopsis* to drought or osmotic stress through improving ROS clearance by reducing H_2_O_2_, increasing proline, and activating antioxidant enzymes (Yang et al., 2015).

Flooding restricts gas diffusion, leading to O_2_ deprivation, known as hypoxia. The low oxygen supply causes plants to shift from aerobic respiration to anaerobic fermentation, resulting in the accumulation of toxic substances, such as alcohols and aldehydes. Flooding responses are generally governed by flooding-induced hypoxia signaling and ethylene. However, there is little information on how the sea purslane responds to flooding or why it is able to grow in water or after flooding.

Sensing the soil moisture levels and adjusting root growth toward the moisture is known as hydrotropism. Although drought and flooding are two opposite stresses, emerging evidence suggests that these stresses may be regulated by a cross-talk between signaling pathways, which is known as the counterintuitive hypothesis (Bin Rahman and Zhang, 2016). Ethylene might play a critical role in the regulation of these two stresses. The master regulator of flooding tolerance, *SUBMERGENCE 1* (*SUB1A*), and possibly *SNORKEL2*, is highly upregulated, which mediates flooding and drought tolerance in rice (Bin Rahman and Zhang, 2022). Although no reports are available on the tolerance of *S. portulacastrum* to both flooding and drought, this species might actually be an ideal plant model for the study of the mechanisms underlying co-tolerance.

### Tolerance to toxic metals

Sea purslane plants also tolerate toxic metals. The tolerance mechanisms vary depending on the type of metal (Alsherif et al., 2023). Ghnaya et al. (2005) compared the responses of two halophytes, *S. portulacastrum* and *Mesembryanthemum crystallinum*, to increased concentrations of cadmium (Cd) in a hydropic culture system. The growth of *S. portulacastrum* was significantly less suppressed by Cd compared to that of *M. crystallinum*, while the Cd content in the shoots of *S. portulacastrum* was 50% less than that of *M. crystallinum*. However, the total K content of *S. portulacastrum* was more than double than that of *M. crystallinum*. In addition, the Ca content of *S. portulacastrum* was significantly higher than that of *M. crystallinum.* Another study on Cd and copper (Cu) found that these metals mainly accumulated in the roots of *S. portulacastrum*, concluding that *S. portulacastrum* is not a hyperaccumulator of Cd and Cu (Feng et al., 2018a). A study on Cd and nickel (Ni) found that 50 μM Cd had no significant effect on the biomass production of *S. portulacastrum*, but either 100 μM Ni or 50 μM Cd combined with 100 μM Ni dramatically affected plant growth. The citrate concentrations decreased in the roots, but increased in the xylem and shoots due to treatments with Cd and Ni, suggesting that citrate may chelate with Cd or Ni, thus contributing to metal tolerance (Mnasri et al., 2015). Fourati et al. (2020) reported that *S. portulacastrum* grown in nutrient solutions with a Ni concentration greater than 50 μM showed chlorosis of the leaves, which was caused by the accumulation of Ni in the photosynthetically active chlorenchyma. In addition, *S. portulacastrum* was found to be more tolerant to lead (Pb) than *Brassica juncea* (Zaier et al., 2010), as Pb(NO_3_)_2_ at 200 μM had no effect on the dry weight accumulation of *S. portulacastrum*, but significantly reduced the dry weight of *B. juncea*. Pb was largely accumulated in the roots of both species, but *S. portulacastrum* had a high level of Pb in shoots, indicating that Pb can be transported to the shoots (Ghnaya et al., 2013). Some reports suggest that the tolerance of *S. portulacastrum* to toxic metals is related to its accumulation of Na (Mariem et al., 2014; Kulkarni et al., 2021). A high level of Na in *S. portulacastrum* could reduce the uptake of metals due to antagonism or a change in the ion homeostasis in plant cells, which results in more tolerance to toxic metals. Further research is warranted to test these propositions.

### Responses to pest and pathogens

*S. portulacastrum* has been shown to tolerate pathogen infection and insect pest attack, which are attributed in part to the high concentration of NaCl in the plant. Thus far, two foliar diseases—leaf blight caused by *Bipolaris sesuvii* (Zhang and Li, 2009) and leaf spot caused by *Gibbago trianthemae* (Chen X. et al., 2022)—have been reported in China. In the US, white blister (*Albugo trianthemae*), root rot (*Phymatatrichum omnivorum*), rust (*Puccinia aristidae*), and root-knot nematode (*Meloidogyne marioni*) have been reported, and some fungal pathogens have been found to be associated with *S. portulacastrum* (Lonard and Judd, 1997). However, there is no concrete evidence available regarding the extent of damage. There have been no reports of insect pests in *S. portulacastrum.* The leaves of *S. portulacastrum* are rich in essential oils. These essential oils have shown activities against eight bacterial species, including *Bacillus subtilis*, *Escherichia coli*, *Acetobacter calcoacetica*, *Clostridium sporogenes*, and *Clostridium perfringens*, and four fungal species: *Aspergillus flavus*, *Aspergillus niger*, *Candida albicans*, and *Penicillium notatum* (Magwa et al., 2006). It is possible that these essential oils could serve as biopesticides against a number of insect pests.

### Rhizospheric microbes contributing to stress tolerance

Plant growth-promoting microbes (PGPMs) are known to contribute to the stress tolerance of sea purslane plants. Karunasinghe et al. (2020) isolated 40 endophytic and 19 rhizospheric fungi from the roots of *S. portulacastrum*, of which endophytic isolates of *Aspergillus insulicola* and *Aspergillus melleus* and a rhizospheric isolate of *Aspergillus luchuensis* were used as biocontrol agents for the control of damping off caused by *Pythium aphanidermatum* in cucumber production. Halotolerant endophytes from *S. portulacastrum* have been shown to improve the salt tolerance of *Vigna mungo* L (John et al., 2023). Recently, a novel fungal strain of *Cladosporium* ‘BF-F’ has been isolated from *S. portulacastrum* roots, and subsequent evaluation showed that it was able to promote plant growth by producing indole-3-acetic acid (IAA) and increasing the uptake of N (Yang et al., 2023). With continuous investigation of *S. portulacastrum*, more PGPMs could be isolated and their roles in the mediation of plant tolerance to multiple stresses will be revealed. It is believed that PFPMs could be cost-effective and eco-friendly alternatives to improving crop productivity under high-saline conditions (Lokhande et al., 2013b; Yang et al., 2023).

## Nutritional and pharmaceutical value of sea purslane

Sea purslane is edible due to its fresh, succulent, and tender leaves, as well as its high nutritional value, including mineral elements, amino acids, and vitamins (**Table 1**). The succulent green leaves have a salty taste and are consumed as sea vegetables. It is also rich in secondary metabolites, particularly bioactive compounds with pharmaceutical properties, making *S. portulacastrum* a medicinal plant species as well.

**Table 1:** Summary of the nutritional value of *Sesuvium portulacastrum* leaves detected by different researchers.

Category	Nutrient	Value	Reference
Carbohydrate	Carbohydrate	1.4 g/100 g	Zeng et al., 2017
	Crude fiber	0.95 g/100 g	Zeng et al., 2017
Protein/amino acids	Protein	3.0 g/100 g	Zeng et al., 2017
	Proline	41.6 mg/100 g FW	Joshi, 1981
	Threonine	23 mg/100 g	Zeng et al., 2017
	Valine	35 mg/100 g	Zeng et al., 2017
	Lysine	40 mg/100 g	Zeng et al., 2017
	Isoleucine	26 mg/100 g	Zeng et al., 2017
	Leucine	50 mg/100 g	Zeng et al., 2017
	Phenylalanine	29 mg/100 g	Zeng et al., 2017
Mineral nutrients	Potassium	3.4%	Fourati et al., 2019
	Calcium	0.3%	Fourati et al., 2019
	Magnesium	49.7 mg/100 g	Main and Waldrop, 2020
	Phosphorous	87.0 mg/100 g	Fourati et al., 2019
	Sulfur	85.0 mg/100 g	Fourati et al., 2019
	Sodium	808 mg/100 g	Main and Waldrop, 2020
	Iron	13.5 mg/100 g	Fourati et al., 2019
	Iodine	955.0 μg/100 g	Gathii, 2013
	Selenium	63.0 μg/100 g	Gathii, 2013
	Zinc	1,490.0 μg/100 g	Gathii, 2013
	Vanadium	579.0 μg/100 g	Gathii, 2013
	Chromium	204.0 μg/100 g	Gathii, 2013
Vitamins	β-carotene	680 μg/100 g	Main and Waldrop, 2020
	Vitamin C	6.95 mg/100 g	Main and Waldrop, 2020
	Vitamin K	164 mg/100 g	Main and Waldrop, 2020
	Vitamin B1	0.02 mg/100 g	Main and Waldrop, 2020
	Vitamin B2	0.06 mg/100 g	Main and Waldrop, 2020
	Folate	17.4 μg/100 g	Main and Waldrop, 2020
	Niacin	0.24 mg/100 g	Main and Waldrop, 2020
	Pantothenic acid	0.17 mg/100 g	Main and Waldrop, 2020
	Vitamin E	1.08 mg/100 g	Zeng et al., 2017
	Vitamin PP	0.53 mg/100 g	Zeng et al., 2017
	Vitamin B6	0.09 mg/100 g	Zeng et al., 2017
Ecdysteroids	20-Hydroxecdysone	2.5 mg/kg	Chandrakala and Maribashetty, 2010
Essential oil	*O*-Cymene	32.61%	Magwa et al., 2006
	α-Pinene	14.12%	Magwa et al., 2006
	2-β-Pinene	13.55%	Magwa et al., 2006
	*Trans*-caryophyllene	8.31%	Magwa et al., 2006
	1,8-Cineole	6.79%	Magwa et al., 2006
	Limonene	6.40%	Magwa et al., 2006

FW, fresh weight.

### Sea purslane leaves are highly nutritious

The leaves of *S. portulacastrum* contain approximately 93% water, 1.4% carbohydrates, 3% protein, different essential mineral nutrients, various vitamins, and β-carotene (**Table 1**). Essential minerals have important physiological functions, and a balanced intake of mineral elements is crucial to human health (Gharibzahedi and Jafari, 2017). *S. portulacastrum* contains almost all essential minerals required by humans, including Ca, chloride (Cl), magnesium (Mg), phosphorus (P), K, Na, sulfur (S), boron (B), chromium (Cr), Cu, iodine (I), iron (Fe), manganese (Mn), selenium (Se), zinc (Zn), and vanadium (V) (**Table 1**). Depending on the ecotype and the growth conditions, the concentrations of these elements vary. It is important to note that sea purslane leaves are rich in iodine, an element generally absent in cultivated vegetables. Iodine is essential for the production of thyroid hormones that control metabolism and are required for proper bone and brain development during pregnancy and infancy (Gharibzahedi and Jafari, 2017). The recommended dietary allowance for iodine per day ranges from 90 mg for children to 220 mg for pregnant women (Pehrsson et al., 2022). The consumption of a small amount of sea purslane leaves could provide the required iodine. In addition, *S. portulacastrum* also contains Se in amounts higher than those in common vegetables, such as broccoli, lettuce, and tomato (Zhang et al., 1993). Selenium is a constituent of many selenoproteins that play critical roles in thyroid hormone metabolism, reproduction, DNA synthesis, and oxidative stress protection (Gharibzahedi and Jafari, 2017). Furthermore, sea purslane leaves contain a rather high level of vanadium. Although vanadium has not been proven to be an essential element for humans, it is essential to some animals as a deficiency in vanadium can lead to growth retardation, bone deformation, and infertility (Nielsen and Uthus, 1991). However, caution should be taken when consuming the leaves of sea purslane as a vegetable. Plants grown in toxic metal-contaminated soils should not be consumed because high concentrations of these metals could result in the absorbed metals being transported to the shoots.

Sea purslane contains 16 essential amino acids in appropriate concentrations (**Table 1**) (Joshi, 1981; Zeng et al., 2017). For every 100 g of edible leaves, the total amino acid content is approximately 556 mg, of which the essential amino acids comprise 219 mg. Thus, the ratio of essential amino acids to total amino acid is 39.38%, which is greater than the 36% that is considered as ideal proteins (FAO/WHO, 1973; Chavan et al., 2001). The content of glutamic acid is the highest (72 mg), followed by aspartic acid (56 mg), leucine (50 mg), and arginine (47 mg), but the methionine content is relatively low and cannot be detected (Zeng et al., 2017). In general, the proportions of six essential amino acids—threonine, phenylalanine, leucine, isoleucine, lysine, and valine—in *S. portulacastrum* leaves are higher than the proposed levels of ideal proteins (FAO/WHO, 1973; Zeng et al., 2017). In addition, glutamic acid, aspartic acid, and NaCl can form sodium glutamate (monosodium glutamate) and sodium aspartate, which are important flavorful substances.

Vitamins are essential substances in the human body for maintaining normal life activities. Sea purslane contains numerous vitamins (**Table 1**). The B vitamins, including B1, B2, niacin (B3), pantothenic acid (B5), and folate (B9); vitamin C; vitamin K (phylloquinone); and β-carotene have been detected in the leaves of *S. portulacastrum* (Zeng et al., 2017; Main and Waldrop, 2020). Of these, the content of vitamin K is the highest, with 1.64 mg/g, followed by folate (0.174 mg/g), as measured in *S. portulacastrum* leaves grown in the Mote aquaponic system (Main and Waldrop, 2020). Vitamin K is involved in blood coagulation and may play a role in protecting individuals from osteoporosis. The National Academy of Sciences established an adequate intake of vitamin K of 120 μg/day for men and 90 μg/day for women (Institute of Medicine, 2001). The amount of vitamin K in sea purslane leaves is greater than that in most vegetables, such as carrots, celery, cucumber, lettuce, pepper, and potatoes (Damon et al., 2005). In addition, β-carotene is a safe source of vitamin A. It is known that the intake of a high dose of vitamin A can be toxic, but the availability of β-carotene could allow the body to convert it into the required amount of vitamin A.

### Pharmaceutical value of sea purslane plants

Sea purslane plants contain a wide range of secondary metabolites including alkaloids, betacyanins, essential oils, lignans, phenolics, and triterpenes (Magwa et al., 2006; Chandrasekaran et al., 2011). A distinct characteristic is their production of ecdysteroids (ECs). ECs are steroidal hormones initially isolated from animals for the control of insect molting, but were subsequently isolated from plants and microbes. Based on the natural source, ECs are classified into zooecdysteroids, phytoecdysteroids (PEs), and mycoecdysteroids. Among the PEs, 20-hydroxyecdysone (20-HE) is the most common. Sea purslane plants contain 20-HE up to 2.5 mg/kg (Chandrakala and Maribashetty, 2010). In addition to improving the tolerance of plants to abiotic and biotic stresses, PEs have shown antidiabetic, anticancer, anti-inflammatory, antibacterial, and antifungal activities in mammals (Arif et al., 2022). Wound healing is a major problem associated with diabetes. Omanakuttan et al. (2016) isolated ecdysterone, a representative PE from *S. portulacastrum*, and found that it interacts with nitric oxide synthase in an epidermal growth factor receptor-dependent manner, promoting cell proliferation, spreading, and migration, thus facilitating the wound healing process.

Plant extracts from sea purslane exhibit strong activity against cancer cells. The diethyl ether extract derived from whole plants showed half-maximal inhibitory concentrations (IC_50_) of 289 μg/mL in a breast cancer cell line (MDA-MB-231), 231 μg/mL in a neuroblastoma cell line (IMR-32), and 183 μg/mL in human colon cancer cells (HCT-116) (Chintalapani et al., 2019). Alzheimer’s disease (AD) is a chronic neurodegenerative disorder. Several cholinesterase inhibitors have been approved by the U.S. Food and Drug Association (FDA) for the treatment of AD. The extracts of sea purslane plants exhibited 50% inhibitory effects on total cholinesterase (TChE) and butyrylcholinesterase (BChE) at concentrations lower than 2 mg/mL, which was comparable to that of the standard drug donepezil (Suganthy et al., 2009).

Terpenes, olefins, and some volatile oils have therapeutic effects on fever and scurvy (**Table 1**) (Magwa et al., 2006). The essential oil extracted from *S. portulacastrum* leaves showed notable antimicrobial activity against both Gram-positive and Gram-negative bacteria, as well as fungi, and some have shown antioxidant activities beneficial to human health (Magwa et al., 2006; Alsrari et al., 2020; Sambou et al., 2023). The omega-3 fatty acid content in *S. portulacastrum* leaves is surprisingly higher than that found in any other leafy vegetables. The fatty acid methyl esters (FAME extract) from *S. portulacastrum* leaves have higher saturated fatty acids than unsaturated fatty acids, and the extract has antimicrobial activity against *Aspergillus fumigatus* and *A. niger* (Chandrasekaran et al., 2011; Patel et al., 2019). The crude extract of *S. portulacastrum* can be used as a non-antibiotic and biological therapeutic agent for the control of bioluminescent disease caused by *Vibrio harveyi* in shrimp larval farming practices (Ramalingam et al., 2016; Dineshkumar et al., 2017; Patel et al., 2019). Furthermore, alkaloids, polysaccharides, saponins, steroids, and triterpenes have been used as antiviral agents for the treatment of hepatitis and other diseases (Lokhande et al., 2013a).

### Sea purslane as a vegetable and a medicinal plant

Young shoots of *S. portulacastrum* are harvested from either the wild or cultivated plants and used as a vegetable for human consumption. They are also fodder for domestic animals. As mentioned above, they are used as medicine for the treatment of a number of human diseases or for the control of diseases and insect pests in crop production.

**Figure 3:**
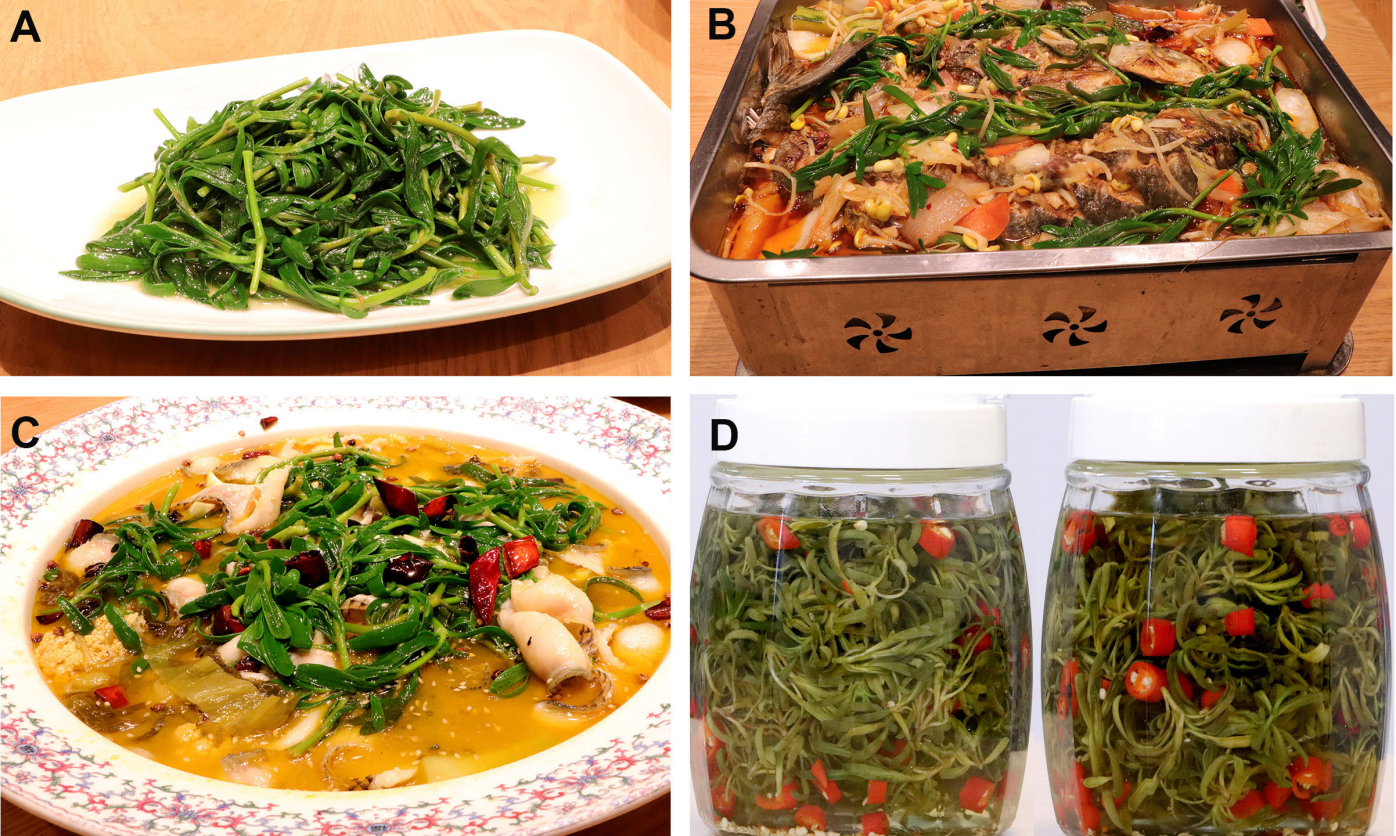
Sea purslane as a leafy vegetable for consumption. **(A)** Cooked sea purslane shoots. **(B, C)** Tender sea purslane shoots used for cooking with fishes **(B)** and with squid **(C)**. **(D)** Pickled sea purslane.

Tender and succulent leaves have a salty taste and have been used as a leafy vegetable in some regions of Africa, Asia, and America (**Table 2**). In Kenya, sea purslane plants are produced in Funzi, Vanga, and Wasini and used as vegetables and medicinal herbs (Gathii, 2013). The leaves of *S. portulacastrum* are cooked and consumed as a salad in Senegal (Mathieu and Meissa, 2007; Sambou et al., 2023). Several ecotypes of *S. portulacastrum* that are able to thrive in diluted seawater have been selected in Hainan, China, and used as sea vegetables (Wang et al., 2022). Young, tender shoots of sea purslane are used as leafy greens, and different recipes have been established for cooking delicious dishes in Fujian, China (**Figure 3**). In Florida, native sea purslane plants have been cultivated with fishes in the Mote Marine Laboratory’s Mote Aquaculture Research Park in Sarasota, and cultivated plants are used as vegetables (Main and Waldrop, 2020). Researchers at Florida Atlantic University’s Harbor Branch Oceanographic Institute studied sea vegetables including *S. portulacastrum* and found that the average edible portion of sea purslane plants is 55%. They can be eaten raw, blanched, sauteed, or cooked as a dish (Galoustian, 2020). It has been documented that *S. portulacastrum* contains druse, a form of calcium oxalate (Cuadra and Cambi, 2017), but there has been no report on its side effects to humans when used as a leaf vegetable. This might suggest that it is similar to spinach, which contains druses, but cooked as a dish or eaten raw as a salad (Kaushal et al., 2022). To assess the safety of sea purslane as an edible vegetable, Yang M. et al. (2021) evaluated the distribution of 15 minerals in the roots, stem, and leaves of plants collected from Dongshan Bay, Fujian Province, China, and found that the contents of Na, K, Ca, and Mg in the leaves were 9.0%, 1.1%, 0.3%, and 0.5%, respectively, while the Cu, Cr, Fe, Mn, Ni, Se, and Zn contents in the leaves were 3.1, 0.4, 61.2, 58.1, 0.3, 0.2, and 9.1 μg/g, respectively. Toxic heavy metals including As, Cd, Hg, and Pb primarily remained in the roots, their contents in the leaves being 0.85, 0.045, 0.011, and 0.15 μg/g, respectively. The authors concluded that toxic metal contamination in the edible portion of sea purslane plants was low and plants are safe for consumption based on the USEPA Risk-Based Concentration Table (Smith, 1995). It should be noted that cooking can alter some of the mineral content in the leaves of *S. portulacastrum*. The cooking process significantly increased calcium and reduced the potassium content (Sambou et al., 2023).

**Table 2:** *Sesuvium portulacastrum* used as food and medicinal plant.

Region	Country	As food	As medicine	Reference
Africa	Kenya (Funzi, Vanga, and Wasini)	Vegetable	Medicinal herb	Gathii, 2013
	Senegal	Salad		Mathieu and Meissa, 2007; Sambou et al., 2023
	Zimbabwe		To treat various infections and kidney diseases	Magwa et al., 2006
	South Africa		To treat various infection and kidney diseases	Magwa et al., 2006
Asia	Bangladesh (Chapai Nawabganj)	Vegetable		Rahman and Khatun, 2020
	China (Fujian, Guangdong, Guangxi, and Hainan)	Leafy vegetable	Medicinal herb	Lokhande et al., 2009; Yang M. et al., 2021; Qiu, 2022
	Philippine	Pickled vegetable called atchara		Hall, 2021
	Southern India	Vegetable	Medicinal herb	Lokhande et al., 2009; Lokhande et al., 2013a
	Pakistan	Vegetable	Medicinal herb	Sajid-ur-Rehman et al., 2021
	Thailand	Leafy vegetable	Folk medicine	Polyium and Phinthida, 2018
North America	USA (Florida, Native Americans)	Leaves eaten raw, cooked, or pickled		Hall, 2021
	USA (Florida)	Salt-loving edible sea vegetable		Galoustian, 2020; Main and Waldrop, 2020

Due to its pharmaceutical value, sea purslane has been used as a medicinal herb to remedy human illness. In South Africa and Zimbabwe, it is used to treat various infections, kidney disorders, and scurvy (Magwa et al., 2006). Plant extracts from the Senegal coast are known to be the best antidote for stings of venomous fish, and leaves have an acidulous flavor of sorrel and are antiscorbutic (Hammer, 1986). Organic extract of *S. portulacastrum* showed anticancer activity against mouse lymphoma cells and hepatic carcinoma (Youssif et al., 2019).

## Sea purslane production and utilization

Soil salinization is one of the major challenges in agriculture. Based on the FAO, 424 million hectares of topsoil (0–30 cm) and 833 million hectares of subsoil (30–100 cm) are affected by salinity (FAO, 2021). The increase in salinization raises an alarm on the global food production. Climate changes with drought and rise in sea levels, as well as population increase, pose further pressure to food security. The global population will increase from 7.6 billion in 2017 to 11.2 billion in 2100 (United Nation, 2017). Concurrently, global hunger has risen, reaching an estimated 821 million in 2017 (Molotoks et al., 2021). Thus, the revitalization of saline soils will be a viable solution to increase crop production, and the use of salt-loving plants, such as *S. portulacastrum*, could improve food production and contribute to the revitalization of saline soils.

### Propagation and production

Due to their tolerance to salinity, drought, and flooding, sea purslane plants can be produced in saline soils and coastal sandy soils as leafy vegetables. Soil salinity is measured based on the total dissolved solids (TDS) or electrical conductivity (EC). A soil with an EC value greater than 4 dS/m is considered to be saline (Marschner, 2011). Saline soils contain different ions, but are dominated by Na^+^ and Cl^−^. An EC of 4 dS/m is equivalent to 40 mM of NaCl (Marschner, 2011). Studies have shown that *S. portulacastrum* can grow in soils with 100 mM NaCl (Slama et al., 2008) and has an EC of 13.04 dS/m (Ramaswamy et al., 2017); thus, *S. Portulacastrum* can grow in different saline soils.

The salinity in water ranges from slight salinity (1–3 g/L dissolved salts) to high salinity (10–35 g/L). Ocean water contains approximately 35 g/L salt (USGS, 2018), of which NaCl is the dominant salt, and the Na concentration is approximately 10.76 g/L (Millero, 1974), i.e., 468 mM. *S. portulacastrum* can grow rapidly at salinity levels between 0 and 23 g/L, but its growth is significantly inhibited when the salinity level is above 35 g/L (Messeddi et al., 2001). Wang et al. (2022) showed that sea purslane plants grown in hydroponic culture with a Na concentration of 500 mM had fresh weights comparable to control plants, indicating that the plants can tolerate Na concentration as high as 500 mM. Thus, for sea vegetable production, *S. portulacastrum* can be directly cultivated in coastal seawater if the total dissolved salt of the water is less than 35 g/L. In fact, sea purslane plants have been planted on floating beds and directly grown in coastal seawater in Fujian Province, China. The Mote Marine Laboratory’s Mote Aquaculture Research Park (Sarasota, FL, USA) cultivates sea purslane in brackish water (salinities ranging from 10 to 18 g/L) marine aquaponic system (Main and Waldrop, 2020).

**Figure 4:**
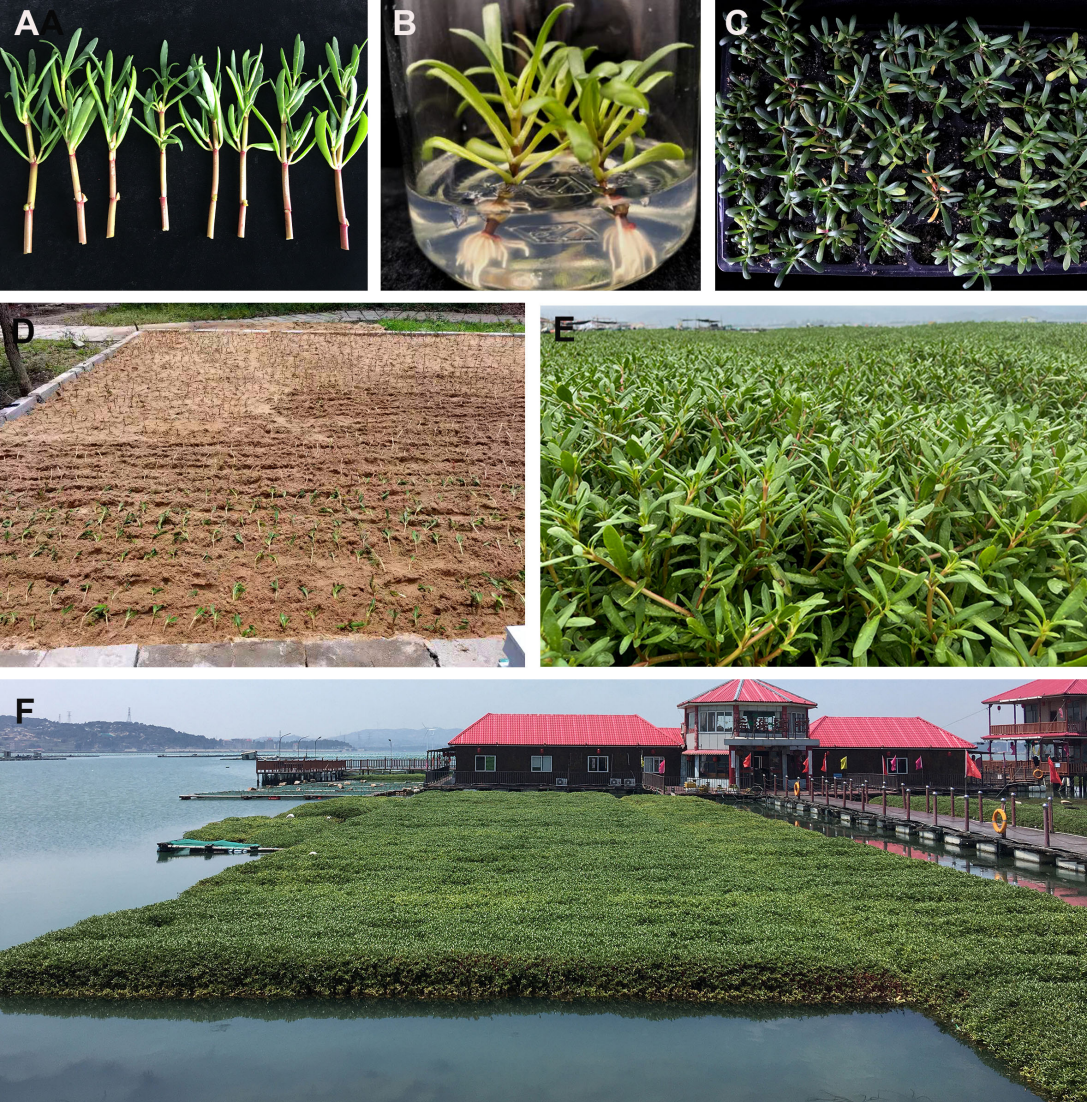
Production of *Sesuvium portulacastrum* in saline soil and water. **(A)** Stem cuttings ready for rooting. **(B)** Plantlets produced through *in vitro* shoot culture. **(C)** Rooted cuttings or liners from *in vitro* culture grown in a plug tray. **(D)** Transplanting rooted cutting to saline soils for sea purslane vegetable production. **(E)** Sea purslane with green, tender, and abundant shoots produced and ready for harvesting. **(F)** Use of sea purslane for the removal of N and P from a contaminated beach.

Sea purslane production starts from seeds, stem cuttings (**Figure 4A**), or plantlets from *in vitro* tissue culture (**Figure 4B**). The cuttings or plantlets are rooted in soilless substrates in plug trays, which are known as liners (**Figure 4C**). Liners can be planted in the soil (**Figures 4D, E**) or in floating beds in water (**Figure 4F**). Propagation using seeds is limited because there is no commercial supply of seeds (Lokhande et al., 2013a). Furthermore, *S. portulacastrum* is an open-pollinated plant, and plants propagated from seeds may not be true to type. Therefore, *S. portulacastrum* is commonly propagated using stem cuttings. Rooted stem cuttings (liners) can be planted in sandy soils and in floating beds to produce healthy plants. However, cutting propagation requires a large number of stock plants and could carry and spread diseases, resulting in large-scale infections and deaths (Zhang and Li, 2009; He et al., 2021; Chen X. et al., 2022). Micropropagation is the most effective method for the rapid propagation of disease-free propagules on a year-round basis (Chen and Henny, 2008). Plantlets derived from *in vitro* shoot culture are healthy and true to type and grow vigorously in plug trays as liners for transplanting (He et al., 2021). Similarly, tissue cultured liners can be planted in saline soils or floating beds. If transplanted in soils, fertilizers containing nitrogen can be applied to improve growth. The tender leaves and stems (shoots) are pinched out from plants as a leafy vegetable. Continuous growth of plants provide more new shoots for harvesting.

### Use of sea purslane for soil desalinization

Sea purslane plants are able to take up a substantial amount of Na from saline soils, transporting it to shoots. Rabhi et al. (2010) reported that *S. portulacastrum* was able to accumulate 872 mg Na in the shoots, which is equivalent to 1 ton/ha. Thus, the plant can be used for the remediation of saline soils through the absorption of Na from the soil, a process known as phytoextraction (Huang et al., 1997). Through the continued cultivation of sea purslane plants on saline soils and the harvest of entire plants, the Na in the soil could be substantially reduced. Sea purslane roots are often associated with PGPMs, and the use of PGPMs will further enhance salt extraction. As a result, the desalinized soils can be used to cultivate other crops.

### Sea purslane for the bioremediation of contaminated soils and water

Toxic heavy metal stress is considered a challenging and emerging threat to sustainable agricultural development. These pollutants are characterized by their persistence in the environment and their highly toxic effects to all living organisms (Wong et al., 2002; Rajkumar et al., 2009). Heavy metals in the soil are absorbed by the roots and can be transported to the shoots depending on the metal and the soil metal concentrations, which could significantly affect many physiological and molecular processes and plant growth. Several metals, including As, Cd, Hg, and Pb, are nonessential to plants and are toxic at low concentrations, mainly through their high affinity for S and N atoms in the amino acid side chain of enzymes or structural proteins (Wei et al., 2003). Moreover, they can compete for nutrients such as Ca, Fe, and Mg for transporters in cell membranes (Carrier et al., 2003). *S. portulacastrum* can absorb Cd, Cu, and Pb in the roots and limit their transport to the shoots. Thus, it could be a candidate for the phytoremediation of metal-polluted saline soils (Ayyappan et al., 2016; Wali et al., 2016; Feng et al., 2018a, Feng et al., 2018b; Fourati et al., 2019; Uddin et al., 2020; Kulkarni et al., 2021). To reiterate, by planting and harvesting sea purslane in metal-contaminated saline soils, metals and Na could be largely removed and the soils be eventually ready for the cultivation of other food crops.

Eutrophication is a serious environmental problem and is expected to increase by 20%–100% in 2050 and by 120%–390% in 2100 according to climate and population forecasts (FAO, 2018). Many coastal waters are severely eutrophic, and it is necessary to reduce the nutrient concentrations in order to meet certain water quality standards. *S. portulacastrum* can be used for the phytoremediation of N and P pollutants from waterbodies, and the biomass produced during this process can be used to feed animals (Lokhande et al., 2013a; Boxman et al., 2017; Liu et al., 2019). *S. portulacastrum* grown in floating beds can significantly reduce dissolved inorganic N and P to meet water quality standards (Liu et al., 2019). Sea purslane plants grown in floating beds not only absorb N and P but also provide fish with food (Senff et al., 2020).

## Conclusion and perspectives

*S. portulacastrum* has been shown to be a unique halophyte species. This plant is tolerant to salt, drought, flooding, and heavy metal stresses. Salt tolerance is the ability to absorb a large amount of Na and then transport Na from the roots to the shoots. The absorption and the accumulation of Na do not affect plant growth, unless the Na concentrations in the growth media are greater than 500 mM. Sea purslane can grow in dry sandy soils and is able to quickly recover from severe drought stress. In addition, this plant can sustain growth after being flooded. It also tolerates heavy metal stress. This tolerance is largely attributed to the limited transport of metal from the roots to the shoots. Because of these characteristics, sea purslane can be used for desalination through the extraction of Na from saline soils. With continued production of sea purslane in saline soils and the harvest of entire plants, the Na in saline soils could be reduced. Desalinated soils can then be used for the cultivation of economically more important food crops. To better utilize sea purslane for the bioremediation of saline soils, the molecular mechanisms behind its tolerance to salt, drought, and flooding stresses should be explored. A better understanding of its multiple tolerances will provide valuable information for improving its production and utilization.

Sea purslane is nutritious and is rich in bioactive compounds. The fleshy and tender shoots are leafy vegetables that can be eaten as a salad, cooked, or pickled. The leaves of *S. portulacastrum* contain high levels of the essential elements iodine and Se. However, the mechanism of the absorption and transport of iodine and Se in sea purslane needs to be further investigated. The plant also produces ECs, but further studies are required to determine how they are synthesized and how they can be better utilized. *S. portulacastrum* has been used as a medicinal plant, and its extracts have shown antioxidant, anti-inflammatory, and anticancer activities, indicating that this plant might contain important compounds that have not yet been identified. Thus, screening and identifying bioactive compounds are also needed.

*S. portulacastrum* is an open-pollinated species. There have been no reports on its breeding. Future efforts should also be placed on germplasm collection, characterization, and utilization. The breeding of sea purslane for enhanced tolerance to abiotic and biotic stresses and for the increased accumulation of bioactive compounds should be highly emphasized. Both traditional breeding and gene editing technologies can be used for its improvement. Although *S. portulacastrum* production is more suitable for tropical and subtropical regions, it can be used in other regions in the summer. To summarize, sea purslane, as a representative halophyte, has great potential for human consumption and for use in the bioremediation of saline soils and eutrophic waterbodies.

## Author contributions

WZ: Data curation, Investigation, Validation, Visualization, Writing – original draft, Writing – review & editing. DW: Data curation, Formal analysis, Methodology, Software, Visualization, Writing – review & editing. DC: Data curation, Formal analysis, Investigation, Software, Visualization, Writing – review & editing. JC: Conceptualization, Investigation, Supervision, Validation, Visualization, Writing – original draft, Writing – review & editing. XW: Conceptualization, Formal analysis, Funding acquisition, Project administration, Resources, Supervision, Writing – review & editing.
